# Cellular Uptake and Antitumor Activity of DOX-hyd-PEG-FA Nanoparticles

**DOI:** 10.1371/journal.pone.0097358

**Published:** 2014-05-14

**Authors:** Wei-liang Ye, Jiang-bo Du, Bang-le Zhang, Ren Na, Yan-feng Song, Qi-bing Mei, Ming-gao Zhao, Si-yuan Zhou

**Affiliations:** 1 Department of Pharmaceutics, School of Pharmacy, Fourth Military Medical University, Xi'an, China; 2 Department of Pharmacology, School of Pharmacy, Fourth Military Medical University, Xi'an, China; 3 West Changle Sanatorium for Xi'an Army Retired Cadres of Fourth Military Medical University, Xi'an, China; University of Sassari, Italy

## Abstract

A PEG-based, folate mediated, active tumor targeting drug delivery system using DOX-hyd-PEG-FA nanoparticles (NPs) were prepared. DOX-hyd-PEG-FA NPs showed a significantly faster DOX release in pH 5.0 medium than in pH 7.4 medium. Compared with DOX-hyd-PEG NPs, DOX-hyd-PEG-FA NPs increased the intracellular accumulation of DOX and showed a DOX translocation from lysosomes to nucleus. The cytotoxicity of DOX-hyd-PEG-FA NPs on KB cells was much higher than that of free DOX, DOX-ami-PEG-FA NPs and DOX-hyd-PEG NPs. The cytotoxicity of DOX-hyd-PEG-FA NPs on KB cells was attenuated in the presence of exogenous folic acid. The IC_50_ of DOX-hyd-PEG-FA NPs and DOX-hyd-PEG NPs on A549 cells showed no significant difference. After DOX-hyd-PEG-FA NPs were intravenously administered, the amount of DOX distributed in tumor tissue was significantly increased, while the amount of DOX distributed in heart was greatly decreased as compared with free DOX. Compared with free DOX, NPs yielded improved survival rate, prolonged life span, delayed tumor growth and reduced the cardiotoxicity in tumor bearing mice model. These results indicated that the acid sensitivity, passive and active tumor targeting abilities were likely to act synergistically to enhance the drug delivery efficiency of DOX-hyd-PEG-FA NPs. Therefore, DOX-hyd-PEG-FA NPs are a promising drug delivery system for targeted cancer therapy.

## Introduction

Doxorubicin is a widely used anticancer agent, but its toxicity to normal tissue and inherent multidrug resistance effect remain as major problems to be solved [1], [2]. Tumor specific nanoparticles are a promising and reliable approach to deliver antitumor drug to the site of action to get the maximum therapy index with the minimum side effects [3]–[7]. Moreover, it is suggested that nanoparticles may be able to bypass p-glycoprotein mediated drug resistance and result in high intracellular drug concentrations [8]–[10]. Thus, various nanoparticles are utilized to selectively deliver anticancer agents to the tumor sites. Some of them, such as Doxil (doxorubicin liposomal) and SMANCS (Zinostatin Stimalamer) [11], [12], have been successfully used in the clinic. Although passive targeting drug delivery systems form the basis of clinical therapy, a number of them can not efficiently diffuse into the tumor tissue [13]. Therefore, a targeting system with high drug delivery efficiency is needed.

The folate receptor is significantly upregulated in many cancer cells and is lowly expressed in normal tissues [14]. Folic acid (FA) has a very high affinity for folate receptor (Kd = 0.1 nmol/L), even after conjugation with therapeutic drugs. Thus, folic acid is an attractive ligand for receptor targeted therapeutics [15], [16]. EC-145 is a conjugate composed of desacetylvinblastine monohydrazide linked through a peptide spacer to folic acid, for the potential treatment of folate receptor overexpressing tumors, in particular ovarian and lung cancers. The *in vitro* studies demonstrated that EC-145 selectively bonded to cells that overexpressed the folate receptor, resulting in dose-dependent cytotoxicity. At present, EC-145 is ongoing phase III clinical trials [17]. In our lab, we synthesized a small molecular FA-mediated targeted delivery system for DOX (FA-AMA-DOX), using aminocaproic acid (AMA) as the linker. AMA was conjugated to the targeting moiety FA via an amide bond, and to the antitumor drug DOX via a hydrazone bond. Although FA-AMA-DOX can deliver DOX to folate over-expressed tumor cells, it is a small molecular conjugate and its drug delivery efficiency *in vivo* needs further evaluation [18].

Polyethylene glycol (PEG) is most frequently used polymeric carrier because of its good biocompatibility, high solubility, low immunogenicity and FDA approval for systemic human use [19]. We conjugated DOX with PEG by hydrazone bond (PEG-hyd-DOX) and amide bond (PEG-ami-DOX) to improve the therapeutic index of DOX. The *in vivo* results indicated that pH-triggered PEG-hyd-DOX conjugate delivered DOX to tumor tissue and released free DOX in acidic tumor environment, and anti tumor efficacy of DOX was improved by using PEG-hyd-DOX conjugate [20].

**Figure 1:**
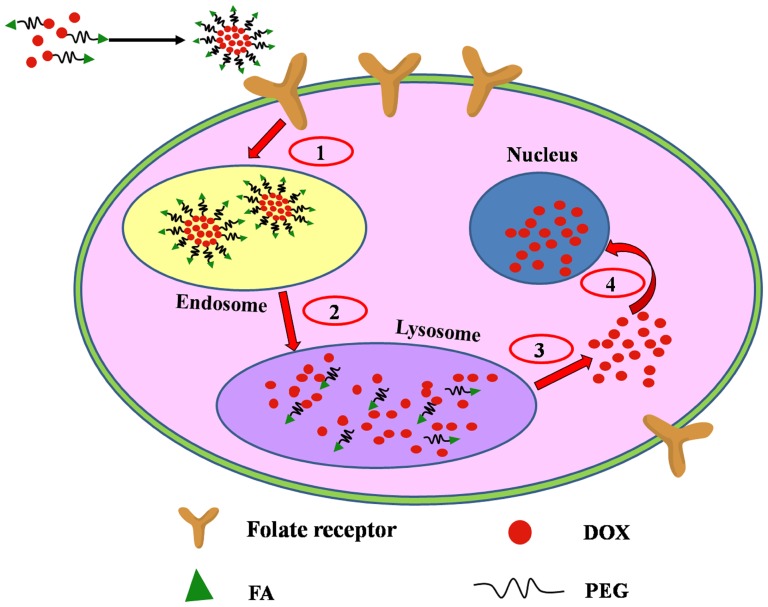
Design of DOX loading DOX-hyd-PEG-FA NPs as an active targeting drug delivery system. (1) FA receptor mediated endocytosis; (2) NPs depolymerization in lysosome; (3) DOX escape from the lysosome; (4) DOX diffuse to nucleus.

In this paper, a pH sensitive polymer DOX-hyd-PEG-FA was synthesized. FA was used as targeting moiety, PEG was used as drug carrier, and DOX was conjugated with PEG through hydrazone bond. In theory, for each DOX-hyd-PEG-FA molecule, only one DOX molecule can enter the cell by one FA receptor mediated endocytosis. When the concentration of DOX-hyd-PEG-FA is increased, the FA receptor will be rapidly saturated. Consequently, the internalization of DOX-hyd-PEG-FA is limited. In order to increase the drug delivery efficiency and antitumor efficacy of DOX-hyd-PEG-FA, DOX-hyd-PEG-FA nanoparticles (NPs) were prepared by using DOX-hyd-PEG-FA as an amphiphilic polymer, and free DOX was encapsulated in DOX-hyd-PEG-FA nanoparticles (as shown in figure 1). In general, after being taken up by tumor cells, DOX-hyd-PEG-FA NPs localized in the lysosome, where pH value is approximately 4.0-6.0 [21], the DOX-hyd-PEG-FA NPs became unstable and were depolymerized [19], [22]. Subsequently, not only DOX encapsulated in the NPs was released in tumor cells, but also large amount of DOX conjugated with PEG also released in tumor cells. Hence, the drug delivery efficiency was greatly enhanced, and the antitumor efficacy of DOX-hyd-PEG-FA NPs was significantly improved.

## Materials and Methods

### 2.1 Materials

Folic acid (FA), N-hydroxysuccinimide (NHS), dicyclohexylcarbodiimde (DCC), trifluoroacetic acid (TFA), and 3-(4,5-dimethylthiaol-2-yl)-2,5- diphenyl-tetrazolium bromide (MTT) were purchased from Sigma-Aldrich (St. Louis, USA). α-Carboxyl-ω-amino poly (ethylene glycol) (HOOC-PEG-NH_2_, average molecular weight is 5000 Da) was purchased from Shanghai Yare Biotech Inc.(Shanghai, China). Doxorubicin was purchased from Hisun Pharmaceutical Co. (Zhejiang, China). All other chemicals were analytical grade and obtained from commercial suppliers without further purification. RPMI1640 medium without folic acid, lysotracker green and 4′,6-diamidino-2-phenylindole (DAPI) were bought from Invitrogen Technologies Company (Carlsbad, USA). KB cells are human epidermal carcinoma cell line from oral cavity on which folate receptor is over-expressed [23]–[25]. A549 cells are human lung adenocarcinoma epithelial cell line on which folate receptor is deficient [23], [24], [26]. HepG2 cells are human liver tumor cell line [27], [28]. KB cell, A549 cell and HepG2 cell were purchased from Institute of Biochemistry and Cell Biology, Chinese Academy of Science, Shanghai, China.

Female athymic nude mice (six weeks old, body weight = 20–23 g) were bought from Experimental Animal Center of Fourth Military Medical University and were allowed water and laboratory chow ad libitum (without folate). A 12-hour light-dark cycle was used. All animal procedures were performed in according with protocols approved by the Animal Care and Use Committee of Fourth Military Medical University (approval date: 18/12/2011, number: 11317).

### 2.2 Preparation of DOX-ami-PEG-FA conjugate

**Figure 2:**
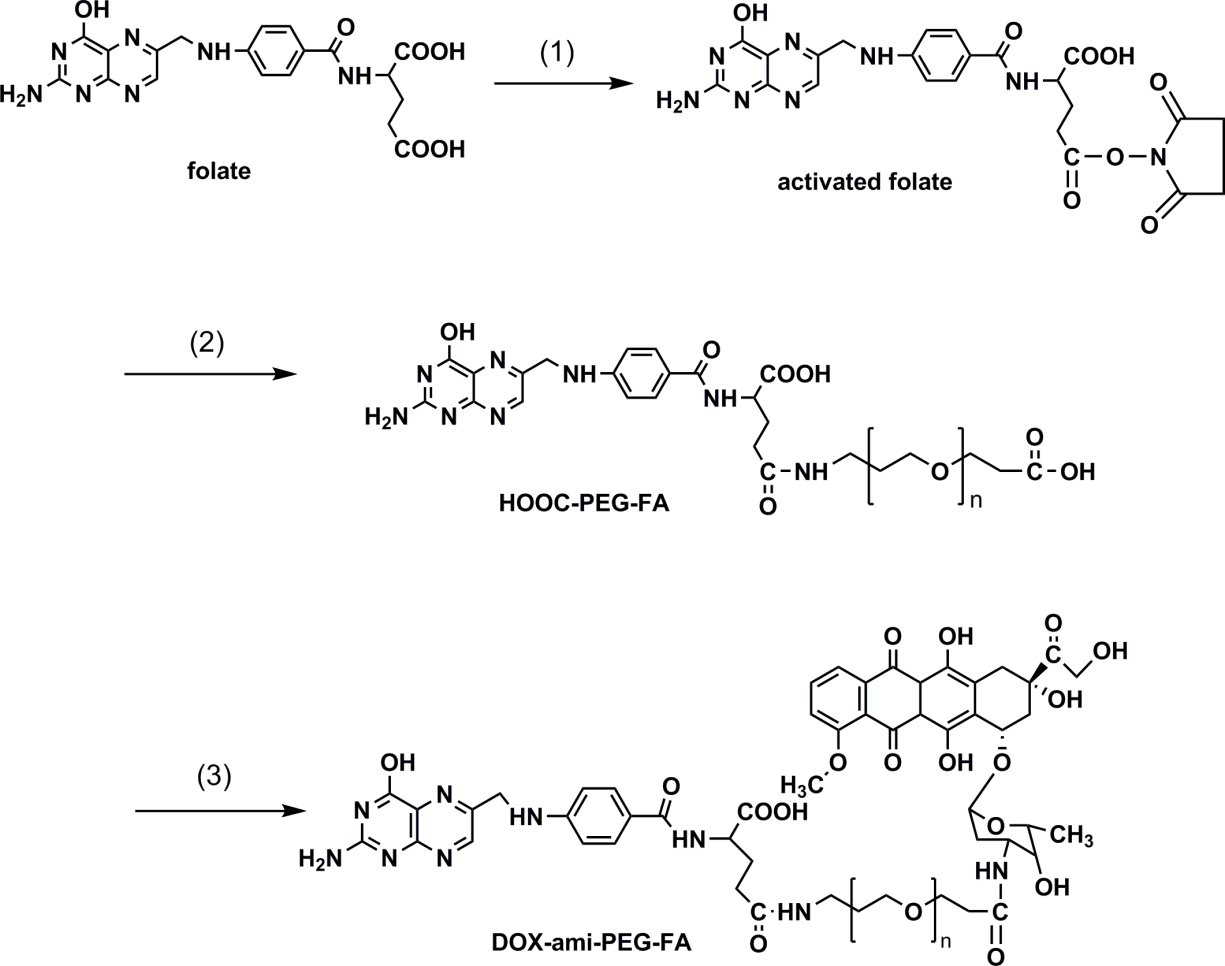
Reaction scheme for the synthesis of DOX-ami-PEG-FA conjugate. (1) NHS, DCC, DMSO; (2) H_2_N-PEG-COOH, TEA, DMSO; (3) NHS, DCC, CH_2_Cl_2_, TEA.

The synthetic scheme for DOX-ami-PEG-FA is shown in figure 2. HOOC-PEG-FA was synthesized as described in the previous study [23]. Briefly, 65 mg of folate (0.15 mmol) dissolved in anhydrous dimethyl sulfoxide (DMSO), and then it was activated by adding dicyclohexyl carbodiimide (DCC, 30.3 mg, 0.15 mmol) and N-hydroxy succinimide (NHS, 16.9 mg, 0.15 mmol) under nitrogen and stired at room temperature for 30 min [29], [30]. The HOOC-PEG-NH_2_ (350 mg, 0.07 mmol) was dissolved in DMSO and added to the folate solution. The reaction was carried out in the presence of triethylamine (TEA) (290 mg, 3.68 mmol) under nitrogen at room temperature for 2 h. Then 5 mL of water and 5 mL of dimethyl formamide (DMF) was added. The compound HOOC-PEG-FA was extracted by chloroform. The organic phase was dried over Na_2_SO_4_ and evaporated. The resulting conjugate was dissolved in water and filtered. The filtered solution was dialyzed against deionized water and lyophilized. Finally, the product was purified by using a Sephadex LH20 column (Pharmacia, Uppsala, Sweden).

350 mg of HOOC-PEG-FA was dissolved in CH_2_Cl_2_, and was reacted with 53 mg of DOX in the presence of 23 mg of NHS, 13 mg of DCC, and 11 mg of TEA. The reaction was performed under nitrogen at room temperature for 4 h. The organic phase was evaporated and the residue was dissolved in water and filtered. The filtered solution was extensively dialyzed against deionized water and lyophilized to obtain DOX-ami-PEG-FA conjugate. The red wine color product was evaluated by HPLC to confirm the absence of unbound free DOX. The productive rate of this step was calculated as the ration between actual yield and theoretical yield of DOX-ami-PEG-FA. In order to synthesize DOX-ami-PEG conjugate without folate, methoxy-PEG-COOH was used instead of HOOC-PEG-FA.

To determine the drug loading of DOX-ami-PEG-FA conjugate, DOX-ami-PEG-FA conjugate was dissolved in 1 mL of 1 mol/L HCl and incubated in a water bath at 50°C for 3 h to yield DOX [31]. The amount of released DOX was analyzed by HPLC with a 2996 photodiode array detector and 2695 pump (Waters Corporation, Milford, MA). A Symmetry C_18_ column (4.6×250 mm, 5 µm; Waters Corporation, Milford, MA) was used. The mobile phase consisted of 70% acetonitrile and 30% H_2_O at a flow rate of 1 mL/min. The wavelength was set to 235 nm. Injection volume was 20 µL. The column temperature was maintained at 25°C. Under this HPLC condition, the free DOX was clearly separated from interference.

### 2.3 Preparation of DOX-hyd-PEG-FA conjugate

**Figure 3:**
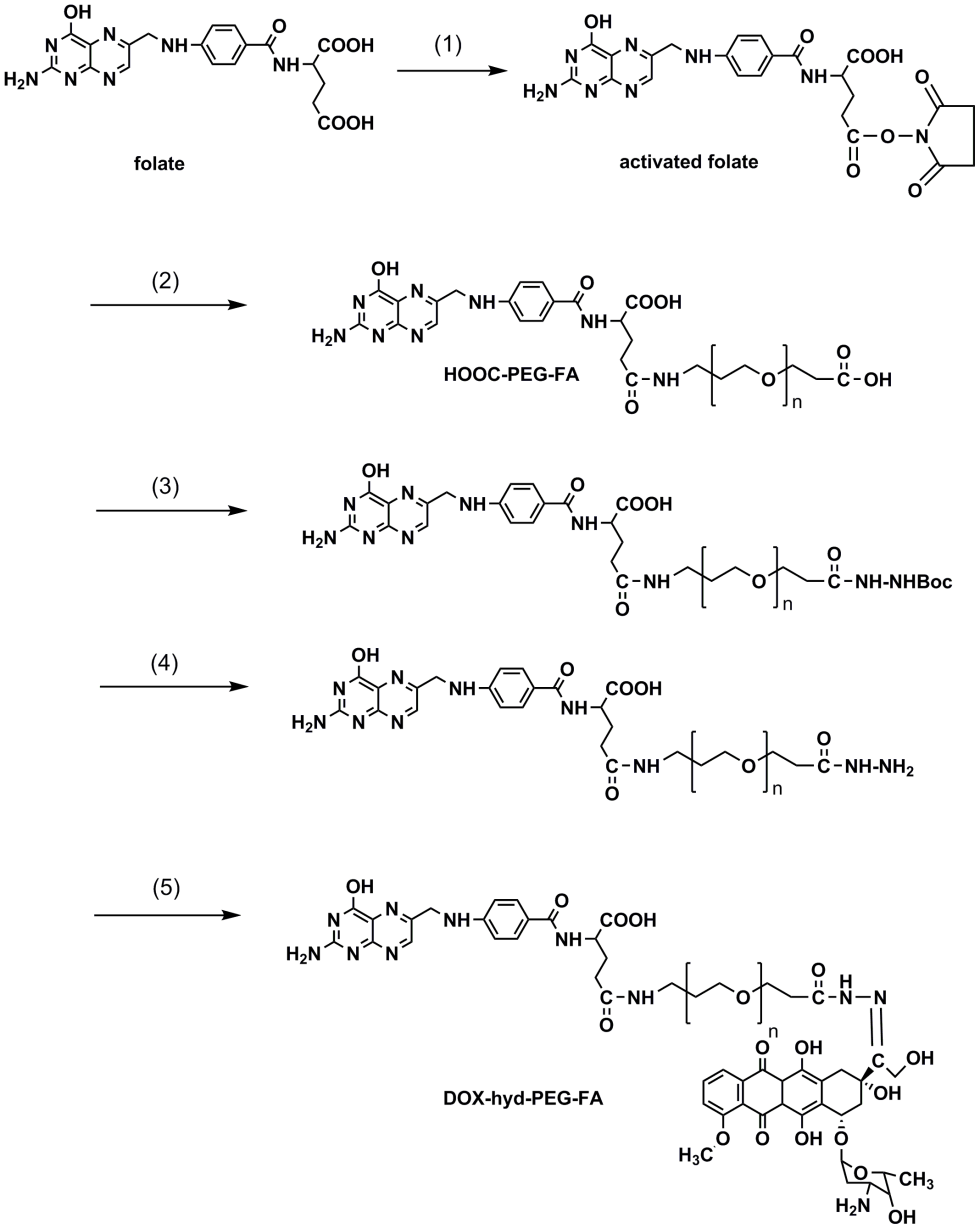
Reaction scheme for the synthesis of DOX-hyd-PEG-FA conjugate. (1) NHS, DCC, DMSO; (2) H_2_N-PEG-COOH, TEA, DMSO; (3) NHS, DCC, BOCNHNH_2_, DMSO, TEA; (4) TFA, DMSO; (5) DOX, TFA, DMSO.

Conjugation of DOX with HOOC-PEG-FA through pH sensitive hydrazone bond was achieved as figure 3 shown [32]. First, Boc-protected hydrazine (Boc-Hyd) was conjugated to HOOC-PEG-FA producing FA-PEG-Hyd-Boc in the presence of DCC and NHS in DMSO [29], [30]. Second, the Boc group in the polymer was removed in 10% trifluoroacetic acid for 20 min to obtain FA-PEG-Hyd. For DOX conjugation, FA-PEG-Hyd (100 mg) was dissolved in 40 mL of DMSO, 20 mg of DOX dissolved in 10 mL of DMSO was then added with trifluoroacetic acid (0.1 mL TFA) as an acid catalyst [18]. The reaction mixture was stirred under nitrogen at room temperature for 48 h and was protected from light. The reaction mixture was diluted with 100 fold volume of water and filtered to protect the formation of micelle. The filtered solution was extensively dialyzed against deionized water and freeze-dried under vacuum to obtain DOX-hyd-PEG-FA conjugate. The red wine color product was evaluated by HPLC to confirm the absence of free DOX. The productive rate of last step of reaction was calculated as the ration between actual yield and theoretical yield of DOX-hyd-PEG-FA. In order to synthesize DOX-hyd-PEG conjugate, methoxy-PEG-COOH was used instead of HOOC-PEG-FA.

To determine the drug loading of DOX-hyd-PEG-FA conjugate, DOX-hyd-PEG-FA conjugate was dissolved in 1 mL of 1 mol/L HCl and incubated in a water bath at 50°C for 3 h to yield DOX. The amount of released DOX was analyzed by HPLC as described above.

### 2.4 Preparation of nanopaticles

The nanopaticles were prepared as previously reported method [33]. Five milligram of DOX-hyd-PEG-FA (or DOX-hyd-PEG,DOX-ami-PEG-FA,DOX-ami-PEG) and 2 mg of free doxorubicin were dissolved in 2 ml of CH_2_Cl_2_, and the mixture solution was added dropwise into 10 ml of deionized water under vigorous stirring. The organic phase was completely evaporated, then centrifuged at 15,000 g for 20 min using CR 21F centrifuge (HITACHI, Japan). After the supernatant was discarded, the nanoparticles were dispersed in water, lyophilized and stored at 4°C until further use. The size, polydispersity index, and zeta potential of nanopaticles were measured by dynamic light scattering (DLS) using a Particle Analyzer (Delsa Nano C, Beckman Coulter).

The drug loading was examined in two methods. (1) Nanoparticles were dispersed in DMSO solution, and the solution was stired for 24 h. The DOX concentration in DMSO solution was detected by HPLC. By this way, the physical drug loading of nanoparticles was calculated. (2) Nanoparticles were dispersed in acetate buffer (pH = 4.0), and the solution was stired for 24 h. The DOX concentration in acetate buffer was detected by HPLC. By this way, the total effective drug loading of nanoparticles was calculated.

### 2.5 Drug release *in vitro*

The *in vitro* drug release study was performed in phosphate buffer solution (pH 5.0, pH 6.5 and pH 7.4). First, 50 mg of NPs was dispersed in 5 mL of release medium and placed in a dialysis bag with a molecular weight cut off of 1 kDa. The dialysis bag was then immersed in 95 mL of the release medium and incubated in a horizontal laboratory shaker at 37°C. Sample (0.5 mL) was periodically collected and the same volume of fresh blank medium was added into the incubation medium. The amount of released DOX was analyzed by HPLC as described above.

### 2.6 Cell culture condition

KB cell, which is a human epidermal carcinoma cell from oral cavity, was maintained in a folate-free RPMI 1640 medium. A549 cell, which is a human lung carcinoma cell, was maintained in a RPMI 1640 medium. HepG2 cell, which is human liver tumor cell line, was maintained in a RPMI 1640 medium. All cell lines were supplemented with 100 units/mL penicillin, 100 units/mL streptomycin, and 10% fetal bovine serum. The cells were cultured as a monolayer in a humidified atmosphere containing 5% CO_2_ at 37°C.

### 2.7 Evaluation of cellular uptake of NPs

DOX itself has fluorescence, it is used directly to measure cellular uptake without additional markers. KB cells (or A549 cells) were seeded into coverglass-containing 24-well plates at density of 100,000 cells/well, incubated at 37°C and grown overnight. DOX or NPs at a concentration of 10 µg/mL equivalent DOX was added and incubated for 4 h at 37°C. Then, the cells were washed with PBS three times and fixed with 1.5% formaldehyde. Coverslip was placed onto glass microscope slide and DOX uptake was analyzed by using a confocal laser scanning microscope (CLSM, Leica, Wetzler, Germany). Digital monochromatic images were acquired using Leica Confocal Software (Version 2.61). To determine the DOX distribution in the nucleus and cytoplasm, the DOX mean fluorescence intensity (MFI) in the CLSM images was measured in a 4 µM^2^ area located in the nucleus or cytoplasm (n = 10 cells) for each sample using ImageJ software.

Cellular uptake of NPs was also monitored semi-quantitatively by using flow cytometry (Coulter XL, Beckman USA). Briefly, KB cells (or A549 cells) were seeded into 6-well plates at a cell density of 1×10^7^ cells/mL in folic acid free medium. After 24 h, the medium was removed, and fresh medium containing 10 µg/mL free DOX or NPs was added (2 mL each well) and incubated with the cells at 37°C for 30 min or 2 h. After incubation for the indicated time, the medium was removed, and cells were washed three times with PBS, then the cells were detached from the plates using trypsin, suspended in PBS and centrifuged for 2 min at 1000×*g* to remove the supernatant, and then the cells were resuspended in 0.2 mL PBS. Cells were finally analyzed by flow cytometry.

### 2.8 Subcellular distribution of DOX

A confocal laser scanning microscopy was used to investigate subcellular distribution of DOX in KB cells after it was incubated with different NPs [34]. Toward this, cells were grown on coverslip to 50% confluence and incubated with NPs (containing 5 µg equivalent/mL DOX) dispersed in culture medium at 37°C for 30 min and 4 h, separately. The cells were then washed five times with PBS and incubated with lysotracker green (50 nmol/L) for 0.5 h. The cells then washed five times with PBS and treated with DAPI (10 µg/mL) for 15 min for nucleus staining. The cells were then washed five times with PBS, fixed with paraformaldehyde for 15 min and stored at 4°C. The cells were imaged by a Zeiss 510 LSMNLO confocal microscope (Carl Zeiss Microscope systems, Jena, Germany) with identical settings for each confocal study.

### 2.9 Cytotoxicity of NPs

The cytotoxicity of NPs against KB cells, A549 cells and HepG2 cells was assessed by using MTT assay. The cells were seeded into 96-well plates and incubated for 24 h. The medium then was replaced with fresh medium containing a series of concentration of NPs and incubated for 48 h. Thereafter, the wells were washed three times with warm PBS and incubated again for another 4 h with FA-free RPMI 1640 containing 5 mg/mL of MTT. After removing the culture medium, 150 µL of DMSO was added to dissolve the precipitate and the resulting solution was measured for absorbance at 490 nm by using a CODA Automated EIA Analyzer (Bio-Rad Laboratories, Hercules, CA).

### 2.10 Animal experiment

Female athymic nude mice (six weeks old, body weight = 20–23 g) were subcutaneously implanted with KB cells (1×10^7^ cells/ 0.1 mL/animal) in rear right flank. There were 6 mice in each group. Treatment was commenced on day 10 post inoculation when tumor size was approximately 80 mm^3^. These tumor-bearing mice were treated with free DOX (5 mg/kg) or NPs (equivalent dose of doxorubicin: 2.5, 5 and 10 mg/kg) by tail vein injection every 7th day (days 1, 7, and 14). Mice were observed daily, and body weight was detected as possible signs of toxicity. Tumor size was measured every 3 days with a caliper in two dimensions and calculated using the formula: volume = LW^2^/2 (L is the long diameter and W is the short diameter of a tumor). On day 42, all surviving mice were euthanized. The heart were removed and fixed in 10% formaldehyde. Formaldehyde-fixed, paraffin-embedded sections were stained with H&E for further histology study.

For distribution study *in vivo*, free DOX and DOX-hyd-PEG-FA NPs were administered to the tumor bearing nude mice via the tail vein with a dosage of 5 mg DOX/kg. Mice were sacrificed at selected time intervals to collect the plasma, organs and tumors, the tissues were rinsed in buffer, weighed and frozen at −20°C until analysis. The amount of DOX in plasma and tissue was measured by HPLC as described above.

### 2.11 Statistical analysis

All data were processed and analyzed by Sigma-Plot 8.0 software. The normality test was done before t-test. The statistical significance was evaluated by t-test and p<0.05 was considered significant.

## Results

### 3.1 Synthesis of DOX-conjugated polymer

**Figure 4:**
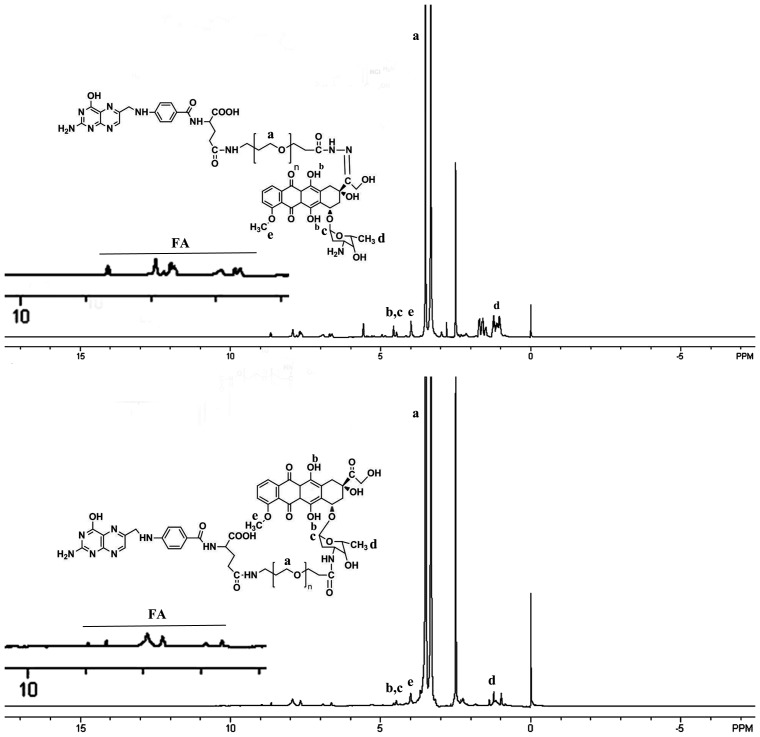
^1^H NMR spectrum of DOX-hyd-PEG-FA (upper panel) and DOX-ami-PEG-FA (lower panel).

**Figure 5:**
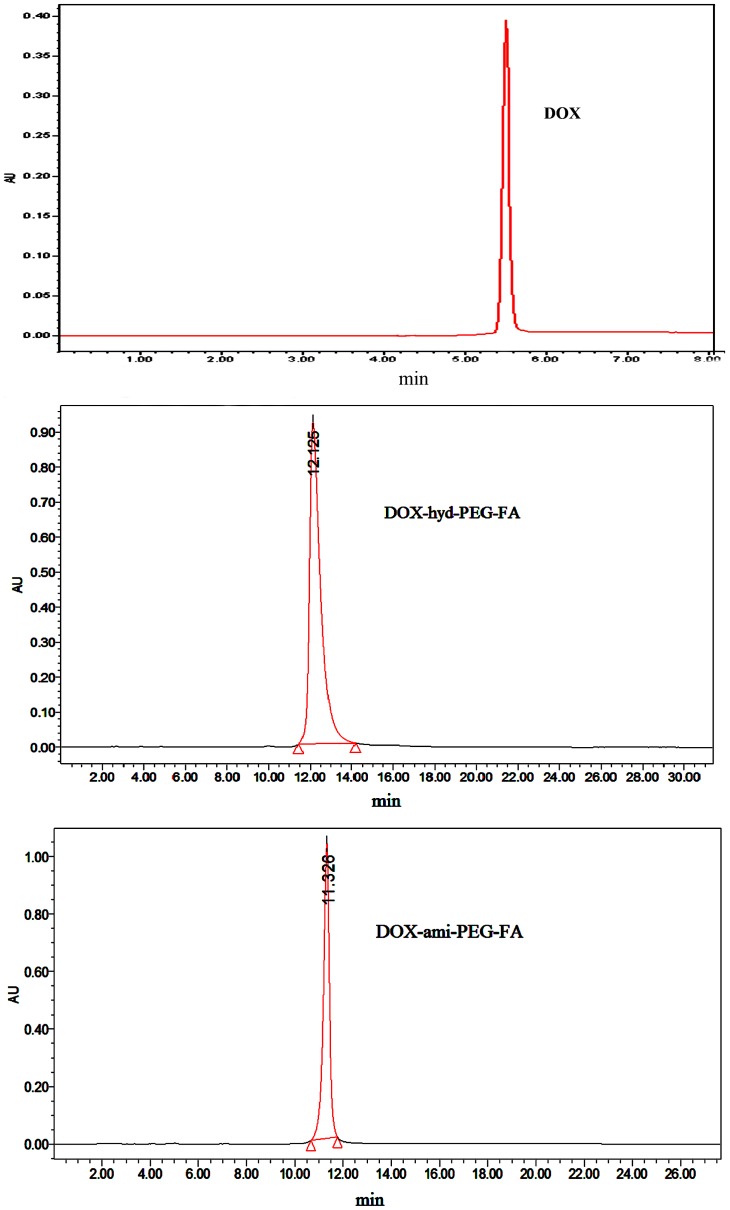
Typical chromatograms of free DOX (upper panel), DOX-hyd-PEG-FA (middle panel) and DOX-ami-PEG-FA (lower panel).

After the product was purified, the yield of DOX-hyd-PEG-FA, DOX-ami-PEG-FA, DOX-hyd-PEG and DOX-ami-PEG was 92%, 89%, 90% and 92% respectively. The DOX conjugated in DOX-ami-PEG, DOX-ami-PEG-FA, DOX-hyd-PEG and DOX-hyd-PEG-FA was 12.8%, 13.2%, 11.7% and 13.6% (w/w) respectively. The ^1^H NMR spectrum of DOX-hyd-PEG-FA and DOX-ami-PEG-FA are shown in figure 4. The presence of FA in DOX-hyd-PEG-FA and DOX-ami-PEG-FA was confirmed by the appearance of signals at 6.7–8.7 ppm in ^1^H NMR spectrum, which corresponded with the aromatic protons of FA. Moreover, the conjugation of DOX was confirmed by the presence of characteristic DOX peaks at 5.4, 4.0 and 2.5 ppm [35]. The PEG backbone was confirmed by the signal at 3.6 ppm [36]. The purity of DOX-hyd-PEG-FA and DOX-ami-PEG-FA was analyzed by HPLC, and typical chromatograms are shown in figure 5, which exhibited single sharp peak at 12.125 min for DOX-hyd-PEG-FA and 11.326 min for DOX-ami-PEG-FA, suggesting that the conjugates were homogeneous without any free DOX.

### 3.2. Characterization of nanoparticles

The size, polydispersity index, zeta potential, drug loading and drug encapsulation efficiency of nanoparticles are shown in table 1. There were no significant differences in zeta potential, particle size polydispersity index, and drug encapsulation efficiency between different NPs. The results also showed that there was not significant difference in the physical drug loading between different nanoparticles. But the total effective drug loading of DOX-hyd-PEG-FA NPs and DOX-hyd-PEG NPs were significant higher than that of DOX-ami-PEG-FA NPs and DOX-ami-PEG NPs.

**Table 1:** Characterization of nanoparticles. Data represented mean±SD (n = 3).

	Particle size (nm)	Zeta potential (mV)	Polydispersity Index	Total effective drug loading^a^ (%)	Physical drug loading^b^(%)	Physical encapsulation efficiency (%)
DOX-hyd-PEG-FA NPs	186±27	−27.6±4.7	0.102±0.028	29.3±3.7**	15.1±1.7	74.3±8.9
DOX-hyd-PEG NPs	188±20	−22.6±3.5	0.181±0.055	27.5±2.5##	14.3±1.8	65.6±6.6
DOX-ami-PEG-FA NPs	213±28	−26.5±5.3	0.167±0.042	14.7±2.1	14.5±1.9	69.4±8.1
DOX-ami-PEG NPs	221±32	−24.6±4.1	0.201±0.053	15.6±2.7	15.2±1.5	64.7±7.7

a : NPs were dispersed in the acetate buffer (pH = 4.0);

b : NPs were dispersed in the DMSO.

**p<0.01 vs DOX-ami-PEG-FA NPs;

## p<0.01 vs DOX-ami-PEG NPs.

**Figure 6:**
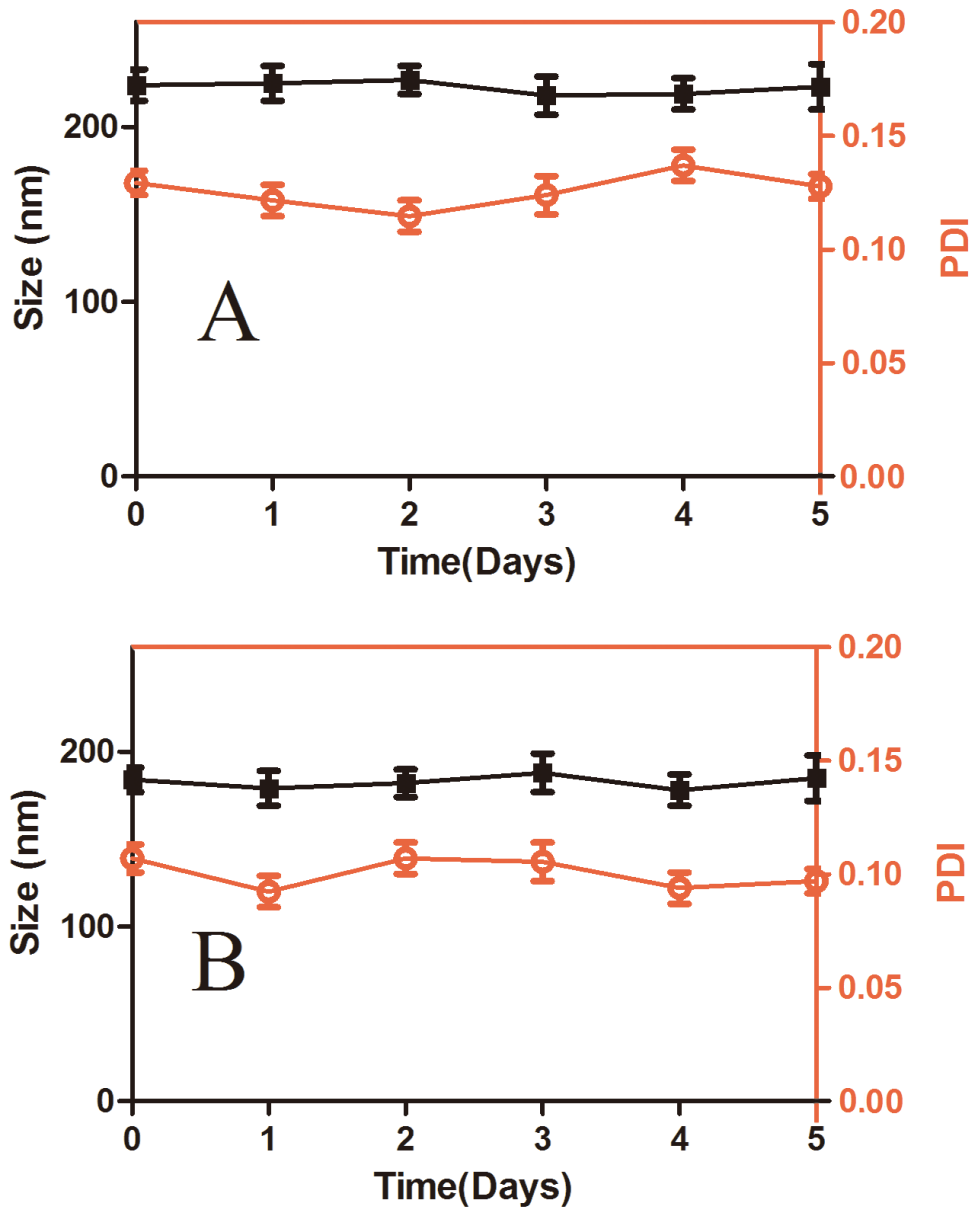
Long-term stability of DOX-ami-PEG-FA NPs (panel A) and DOX-hyd-PEG-FA NPs (panel B) in terms of particle size and polydispersity index (PDI) in PBS at room temperature. n = 3.

The size stability of DOX-hyd-PEG-FA NPs and DOX-ami-PEG-FA NPs in PBS (pH = 7.4) at 37°C is shown in figure 6. The results indicated that DOX-hyd-PEG-FA NPs and DOX-ami-PEG-FA NPs were stable in five days in PBS (pH = 7.4) at 37°C.

### 3.3 Drug release characteristics *in vitro*

**Figure 7:**
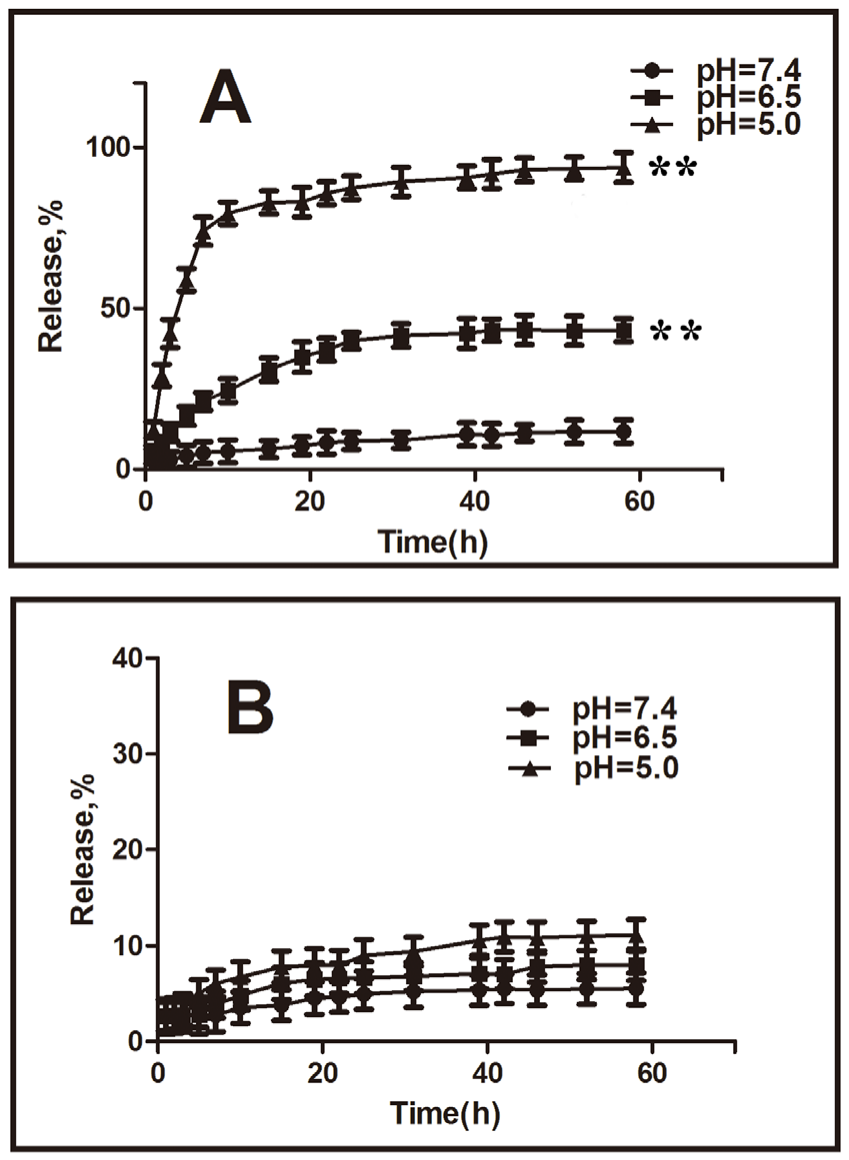
DOX release profiles from the DOX-hyd-PEG-FA NPs (panel A) and DOX-ami-PEG- FA NPs (panel B) in different pH medium. **P<0.01 vs pH 7.4 medium at 58 h, n = 3.

The drug release characteristics of the DOX-hyd-PEG-FA NPs and DOX-ami-PEG-FA NPs were investigated in a neutral environment (pH 7.4) and in an acidic environment (pH 5.0 and 6.5) at 37°C to assess the feasibility of using DOX-hyd-PEG-FA NPs and DOX-ami-PEG-FA NPs as anticancer drug delivery carriers. The results are shown in figure 7. The rate and amount of DOX released from the DOX-hyd-PEG-FA NPs were dependent on the pH of the medium. DOX-hyd-PEG-FA NPs showed a much faster DOX release in pH 5.0 medium than in pH 7.4 medium. In pH 5.0 medium, DOX-hyd-PEG-FA NPs released out 80% of loaded DOX in 10 h, and liberated 94% of loaded DOX in 58 h. However, in pH 7.4 medium, the DOX-hyd-PEG-FA NPs released only 5% of loaded DOX in 10 h and less than 12% in 58 h. The rate and amount of DOX released from the DOX-ami-PEG-FA NPs were independent on the pH of the medium. DOX-ami-PEG-FA NPs released out 5% of loaded DOX in 58 h in pH 7.4 medium, and released out 11% of loaded DOX in 58 h in pH 5.0 medium.

### 3.4 Cellular uptake of NPs

**Figure 8:**
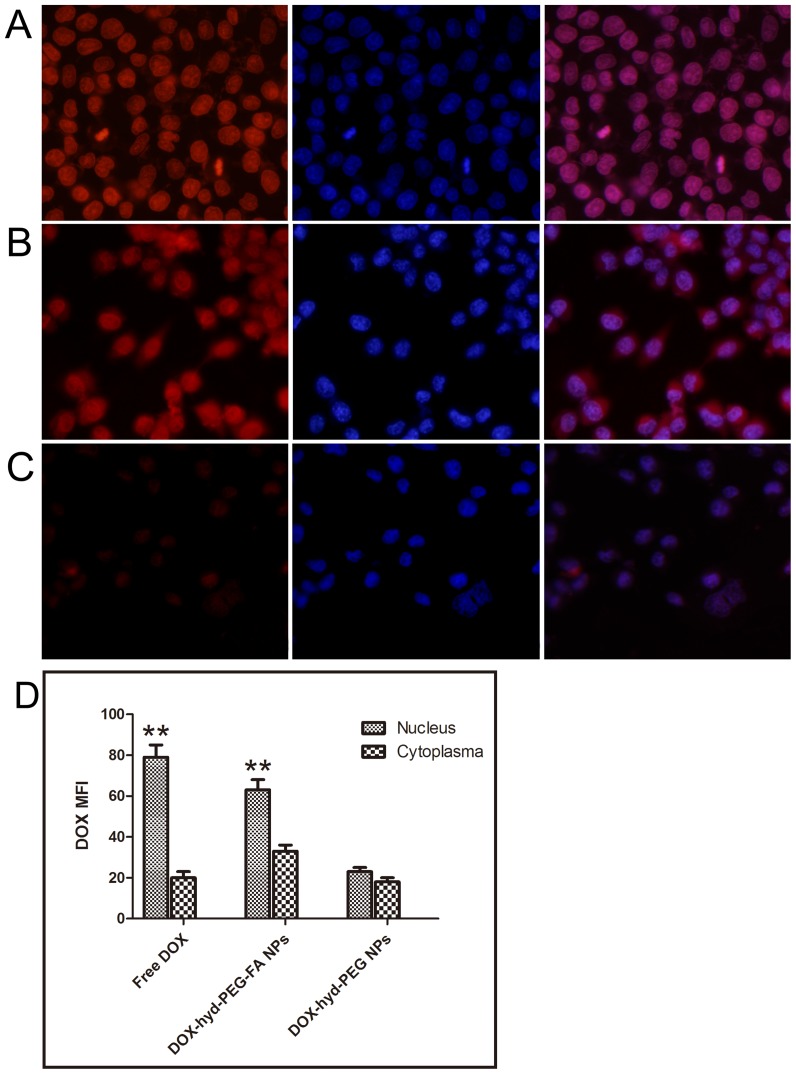
Confocal laser scaning microscopy (CLSM) images of KB cells incubated with DOX (panel A), DOX-hyd-PEG-FA NPs (panel B) and DOX-hyd-PEG NPs (panel C) at 37°C for 4 h. DOX concentration was 10 µg/mL. The pink region shows the localization of DOX (red) in the nucleus (blue). DOX mean fluorescence intensity (MFI) in the nucleus and cytoplasm of the KB cells are shown in panel D. **P<0.01 vs cytoplasma with the same treatment, n = 5.

**Figure 9:**
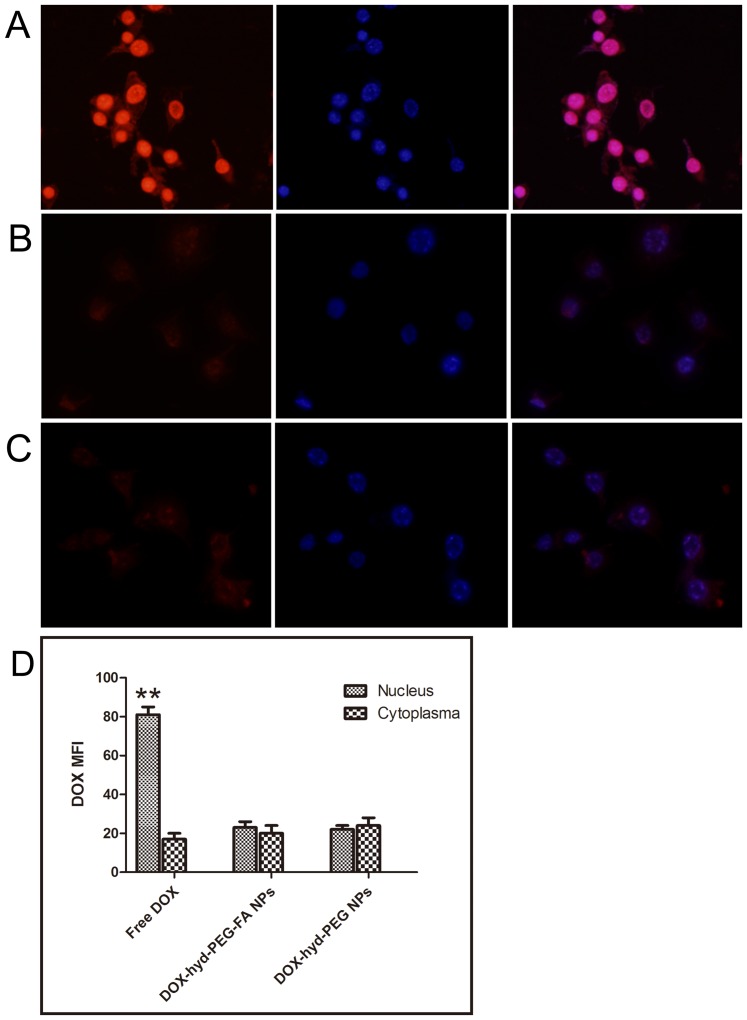
Confocal laser scaning microscopy (CLSM) images of A549 cells incubated with DOX (panel A), DOX-hyd-PEG-FA NPs (panel B) and DOX-hyd-PEG NPs (panel C) at 37°C for 4 h. DOX concentration was 10 µg/mL. The pink region shows the localization of DOX (red) in the nucleus (blue). DOX mean fluorescence intensity (MFI) in the nucleus and cytoplasm of the KB cells are shown in panel D. **P<0.01 vs cytoplasma with the same treatment, n = 5.

After KB cells and A549 cells were incubated with free DOX for 4 h, DOX was predominantly accumulated in the nucleus, which is shown in figure 8A and figure 9A respectively. When KB cells were incubated with DOX-hyd-PEG-FA NPs, large amount of DOX was distributed in the nucleus (figure 8B). Little amount of DOX was distributed in the nucleus after DOX-hyd-PEG NPs were incubated with KB cells (figure 8C). When A549 cells were incubated with DOX-hyd-PEG-FA NPs and DOX-hyd-PEG NPs, little amount of DOX was distributed in the nucleus (figure 9B and figure 9C).

**Figure 10:**
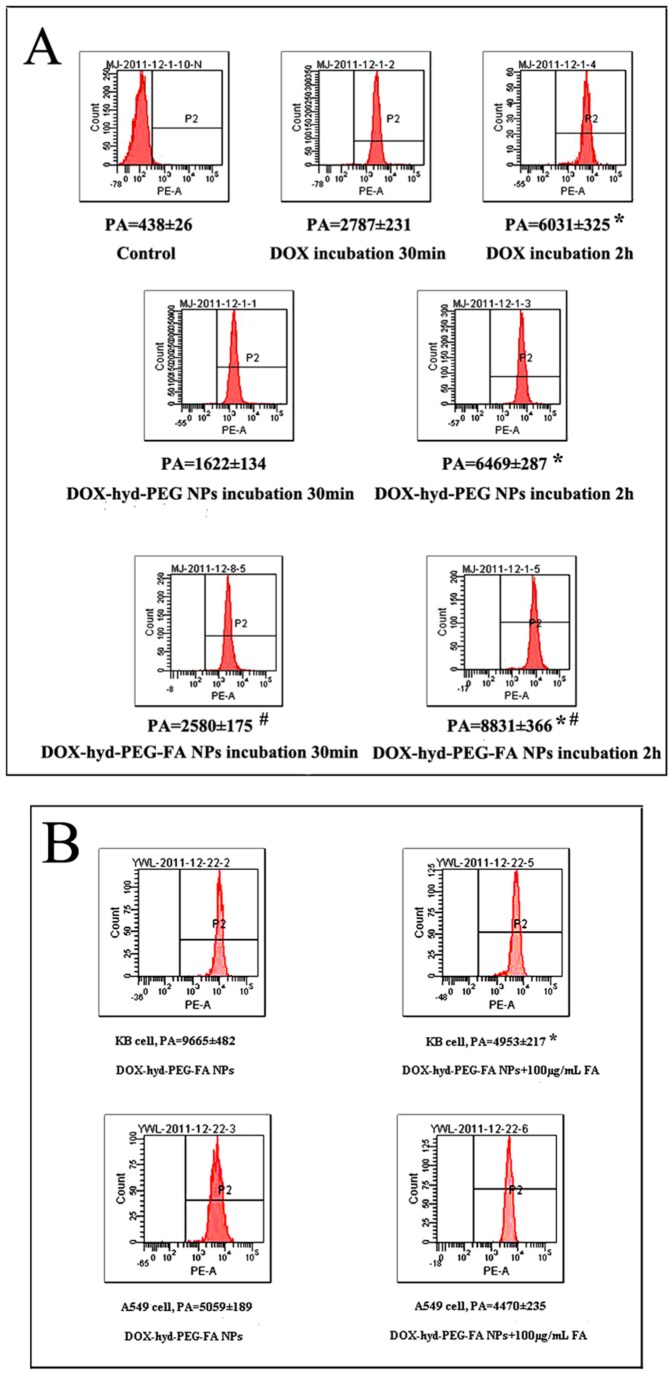
Panel A-Flow cytometry results of KB cells that were incubated with DOX,DOX-hyd-PEG NPs and DOX-hyd-PEG-FA NPs for 30 min and 2 h. DOX concentration was 10 µg/mL. PA-peak area. *p<0.05 vs same treatment at 30 min; ^#^p<0.05 vs DOX-hyd-PEG NPs at the same time point, n = 5. Panel B-Flow cytometric analysis of KB cells and A549 cells treated with DOX-hyd-PEG-FA NPs when exogenous folate was absent and present in the culture medium. DOX concentration was 10 µg/mL.Incubation time was 2 h. PA-peak area. *p<0.05 vs DOX-hyd-PEG-FA NPs on KB cells, n = 5.

The cellular uptake of DOX and NPs were further semi-quantitatively investigated in KB cells and A549 cells by using flow cytometry. The typical pictures and statistical results are shown in figure 10. Cellular uptake of DOX-hyd-PEG-FA NPs in KB cells was increased in time-dependent manner. The intracellular uptake of DOX-hyd-PEG-FA NPs in KB cells was greater than that of DOX-hyd-PEG NPs. When KB cells were incubated with exogenous folate and DOX-hyd-PEG-FA NPs, the uptake efficiency of DOX-hyd-PEG-FA NPs was attenuated obviously. The exogenous folate almost had no effect on the intracellular uptake of DOX-hyd-PEG-FA NPs in A549 cells.

### 3.5 The subcellular distribution of DOX delivered by DOX-hyd-PEG-FA NPs and DOX-ami-PEG-FA NPs

**Figure 11:**
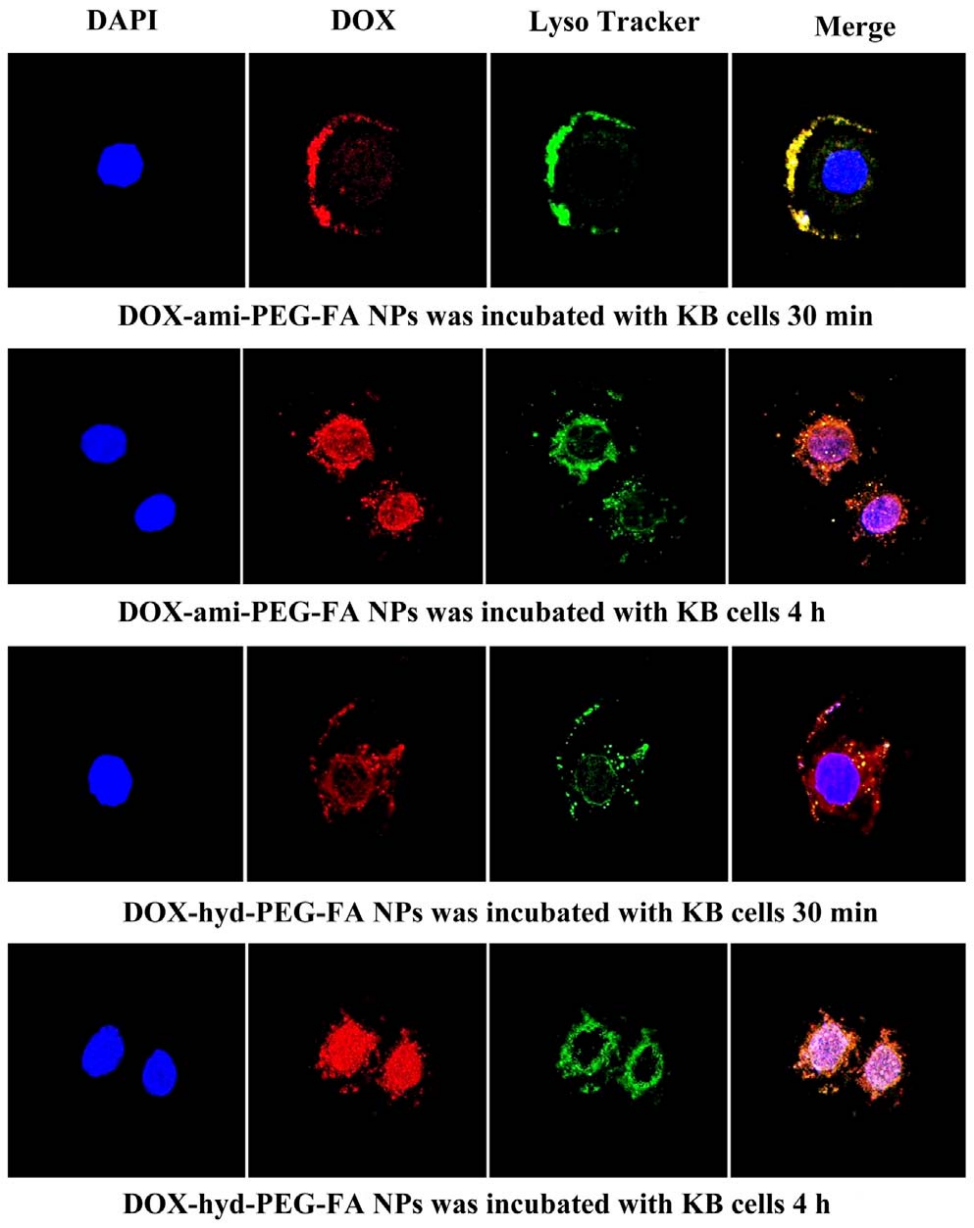
Subcellular localization of DOX in KB cells delivered by DOX-ami-PEG-FA NPs and DOX-hyd-PEG-FA NPs. The pink region shows the localization of DOX (red) in the nucleus (blue), the yellow region shows the localization of DOX (red) in the lysosome/endosome (green).

Distribution of DOX in the nucleus and endosome/lysosome was investigated by triple-labelling with the nucleus-selective dye (DAPI, blue), the fluorescent conjugates (DOX, red), and dyes selective for acidic endolysosomes (LysoTracker green, green). The imaged cells treated with DOX-ami-PEG-FA NPs and DOX-hyd-PEG-FA NPs is presented in figure 11. Both NPs were localized in endolysosomes at early time points. DOX-hyd-PEG-FA NPs showed a predominant nuclear distribution at 4 h in KB cells. When KB cells were treated with DOX-ami-PEG-FA NPs, less DOX was seen in the nucleus, and main fraction of DOX was still found in endolysosomes at 4 h, indicating DOX-ami-PEG-FA NPs were relative stable in endolysosomes.

### 3.6 Cytotoxicity of NPs

**Figure 12:**
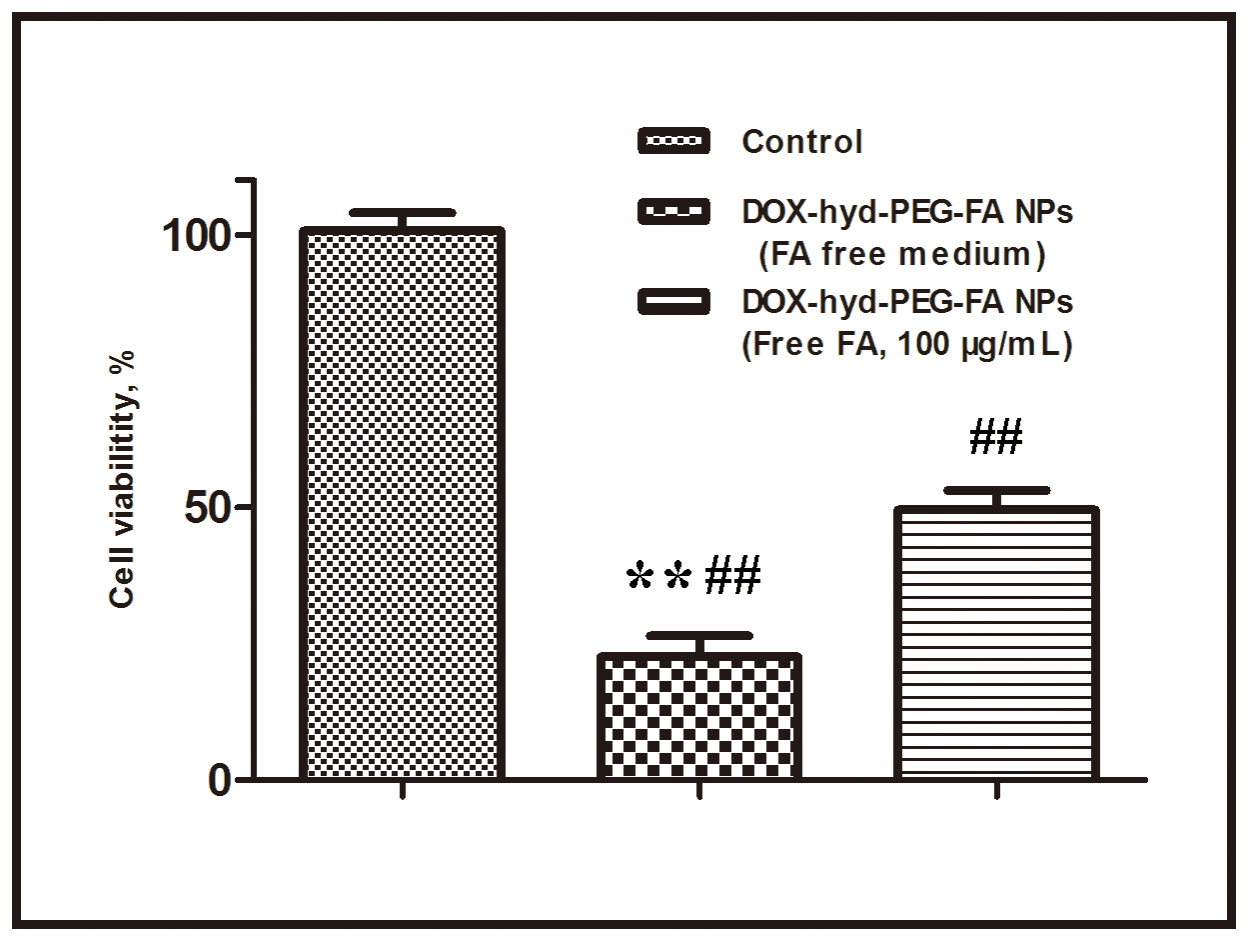
Effect of exogenous free FA on the viability of KB cells incubated with DOX-hyd-PEG-FA NPs for 48 h. DOX concentration was 0.6 µg/mL. **p<0.01 vs DOX-hyd-PEG-FA NPs+FA; ^##^p<0.01 vs control, n = 5.

The cytotoxic effect of free DOX, DOX-hyd-PEG-FA NPs, DOX-ami-PEG-FA NPs, DOX-hyd-PEG NPs and DOX-ami-PEG NPs against the KB cells, A549 cells and HepG2 cells is shown in table 2. The results showed that the toxicity of DOX-hyd-PEG-FA NPs and DOX-hyd-PEG NPs on A549 cells was much higher than that of DOX-ami-PEG-FA NPs and DOX-ami-PEG NPs respectively. There was no significant difference in the viability when A549 cells were treated with DOX-hyd-PEG-FA NPs and DOX-hyd-PEG NPs. For KB cells, the results showed that the cell viability in the presence of DOX-hyd-PEG-FA NPs was much lower than that of free DOX and DOX-hyd-PEG NPs. HepG2 cells showed the similar results with KB cells. To estimate the effect of free FA on the cytotoxicity of DOX-hyd-PEG-FA NPs, KB cells were incubated with DOX-hyd-PEG-FA NPs and 100 µg/mL FA, and the result is shown in figure 12. The cell viability treated with DOX-hyd-PEG-FA NPs was approximately 29% in FA-free medium, but it was about 52% at the presence of 100 µg/mL FA.

**Table 2.** Cytotoxicity for A549 cells, KB cells and HepG2 cells treated with DOX, DOX-hyd-PEG-FA NPs, DOX-hyd-PEG NPs, DOX-ami-PEG-FA NPs and DOX-ami-PEG NPs.

### 3.7 *In vivo* antitumor activity of DOX-hyd-PEG-FA NPs

**Figure 13:**
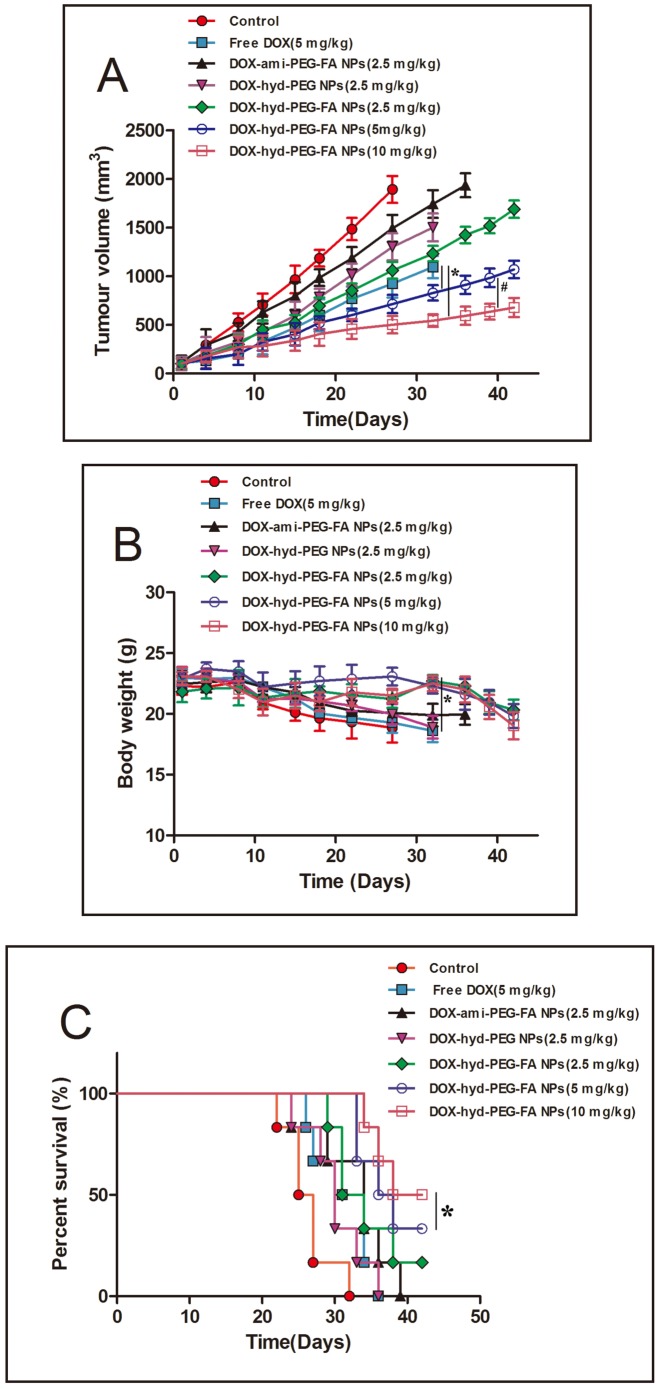
In vivo antitumor activity of DOX-hyd-PEG-FA NPs. Athymic nude mice xenografted with KB cells were intravenously injected with different doses of DOX-hyd-PEG-FA NPs (2.5, 5 and 10 mg/kg equivalent DOX) and one dose of DOX (5 mg/kg) every 7 day (days 1, 7, and 14). Panel **A**-tumor volume changes of tumor bearing mice, *P<0.05 vs free DOX, ^#^P<0.05 vs 5 mg/kg DOX-hyd-PEG-FA NPs, n = 3. Panel **B**-body weight changes in tumor bearing nude mice, *P<0.05 vs free DOX, n = 3. Panel **C**-survival curve of tumor bearing mice. *P<0.05 vs free DOX, n = 3.

In order to further elucidate the role of FA and hydrazone bond in NPs *in vivo*, DOX-hyd-PEG-FA NPs, DOX-hyd-PEG NPs and DOX-ami-PEG-FA NPs were used to treat female athymic nude mice xenografted with KB cells. The *in vivo* anti-tumor activities of DOX-hyd-PEG-FA NPs, DOX-hyd-PEG NPs and DOX-ami-PEG-FA NPs are shown in figure 13. The statistic results of survival are shown in table 3. Overall, compared with same dose of DOX, DOX-hyd-PEG NPs and DOX-ami-PEG-FA NPs treated group, DOX-hyd-PEG-FA NPs treated group showed an improved therapeutic effect in dose-dependent manner in terms of survival rate and tumor growth inhibition. DOX-hyd-PEG-FA NPs treated mice did not show any obvious side effect. The life span of animals treated with DOX-hyd-PEG-FA NPs was significantly longer than that of the animals treated with DOX.

**Table 2:** Cytotoxicity for A549 cells, KB cells and HepG2 cells treated with DOX, DOX-hyd-PEG-FA NPs, DOX-hyd-PEG NPs, DOX-ami-PEG-FA NPsz and DOX-ami-PEG NPs.

Drugs	IC_50_ (µmol/L)		
	A549 cells	KB cells	HepG2 cells
DOX	3.3±0.8	2.2±0.6	2.9±0.4
DOX-hyd-PEG-FA NPs	2.7±0.4*	0.8±0.2** ## $$	1.5±0.3* # $
DOX-hyd-PEG NPs	3.2±0.7	2.8 ±0.3	3.1±0.6
DOX-ami-PEG-FA NPs	4.9±1.1	3.3±0.9	3.8±1.3
DOX-ami-PEG NPs	4.7±0.9	4.0±1.2	4.3±0.8

*p<0.05,

**p<0.01 vs DOX-ami-PEG-FA NPs.

# p<0.05,

## p<0.01 vs DOX-hyd-PEG NPs.

$ p<0.05,

$$ p<0.01 vs DOX.

**Table 3:** Statistic analysis of survival of tumor bearing mice.

Treatment group	Median survival (d)	Mean survival (d)	Maximal survival (d)	*P*
Control	25	26.33±1.63	32	-
Free DOX(5 mg/kg)	31	31.33±1.68	36	0.045^a^
DOX-hyd-PEG-FA NPs (2.5 mg/kg)	31	34.17±1.85	42	0.386^b^
DOX-hyd-PEG-FA NPs (5 mg/kg)	36	37.33±1.52	42	0.049^b^
DOX-hyd-PEG-FA NPs (10 mg/kg)	38	39.00±1.31	42	0.007^b^

a-compared with control, b-compared with free DOX.

### 3.8 Drug biodistribution

**Figure 14:**
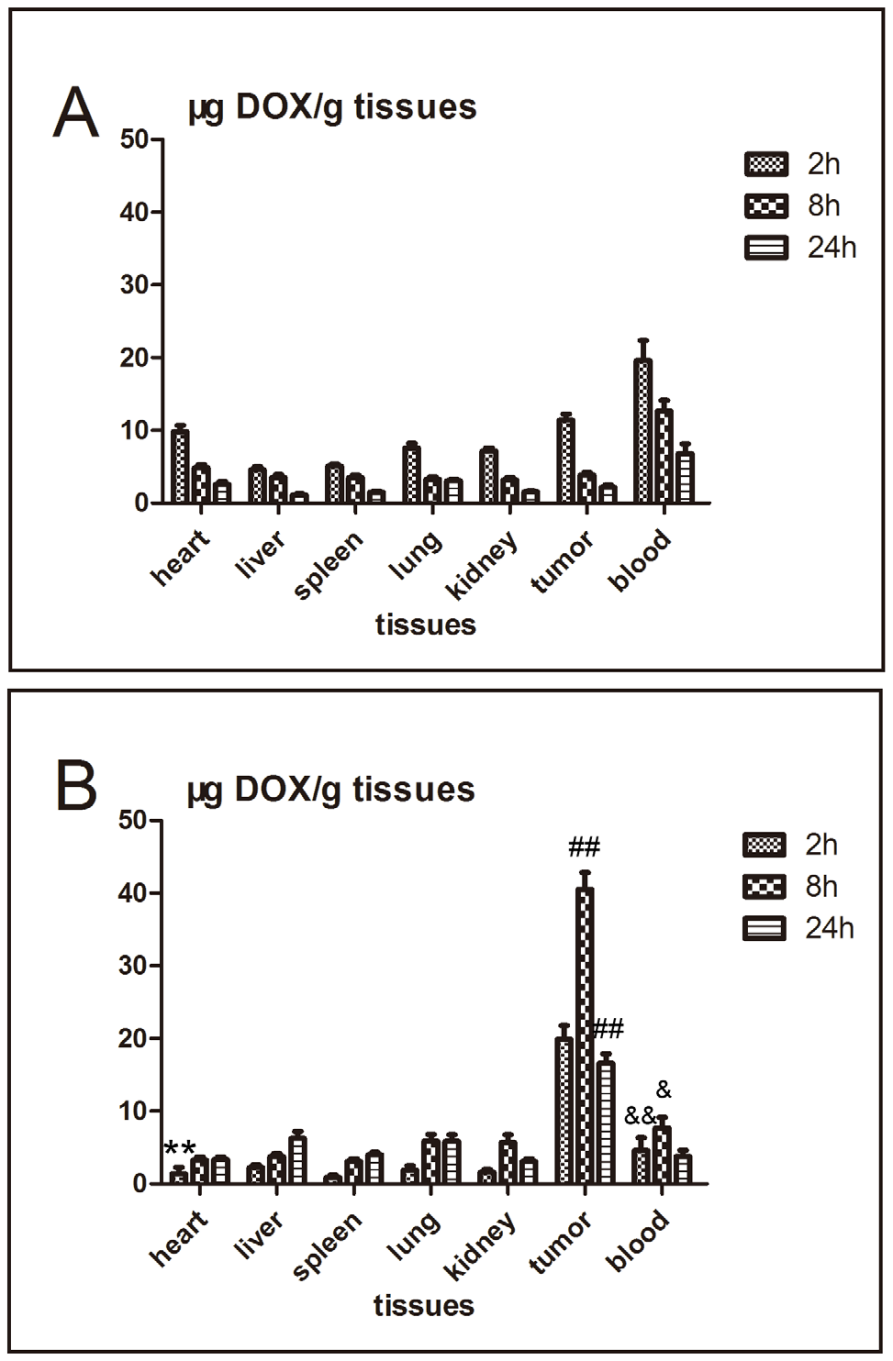
The DOX distribution in tumor bearing nude mice after intravenous injection of either free DOX (panel A) or DOX-hyd-PEG-FA NPs (panel B). **P<0.01 vs free DOX group in heart tissue at the same time point; ^##^P<0.01 vs free DOX group in tumor tissue at the same time point; ^&^P<0.05, ^&&^P<0.01 vs free DOX group in blood at the same time point. n = 5.

The distribution of DOX in tumor bearing nude mice after intravenous injection of free DOX or DOX-hyd-PEG-FA NPs is shown in figure 14. DOX was typically accumulated in liver, heart, spleen, lung, kidneys and tumor after intravenous injection of either free DOX or DOX-hyd-PEG-FA NPs. Compared with free DOX, DOX-hyd-PEG-FA NPs greatly increased the accumulation of DOX in the tumor, and significantly decreased the accumulation of DOX in heart and blood.

### 3.9 Pathological findings

**Figure 15:**
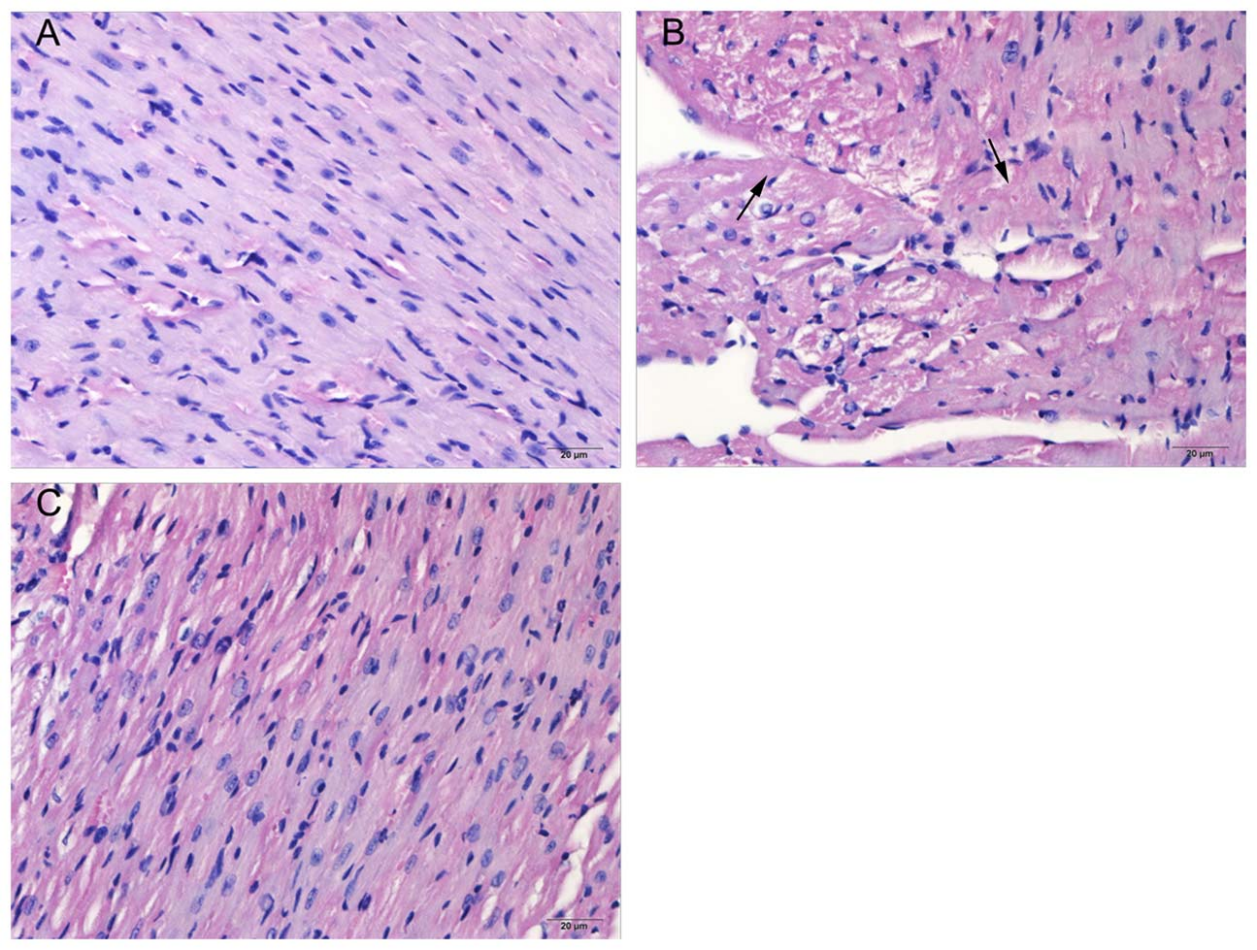
Histological sections of heart from tumor bearing nude mice. Haematoxylin and eosin stain. Panel **A**-negative control mice (tumor bearing mice treated with normal saline). Panel **B**-doxorubicin treated mice (5 mg/kg), degenerative myofibrils (arrows) are seen. Panel **C**-DOX-hyd-PEG-FA NPs treated mice (equivalent dose of doxorubicin:5 mg/kg).

Representative heart section in the control, doxorubicin treated and DOX-hyd-PEG-FA NPs treated groups are shown in figure 15. Structural abnormalities were not found in tumor bearing mice treated with normal saline. Cardiac tissues from doxorubicin-treated animals showed widespread marked structural abnormalities, including cardiomyocyte necrosis, vacuolization and myofibrillar loss. In contrast, necrotic cardiomyocytes, vacuolization and myofibrillar loss were rare in heart tissue from mice treated with DOX-hyd-PEG-FA NPs.

## Discussion

When polymeric NPs are designed and prepared, there are several parameters that need to be taken into consideration, such as particle size, drug loading, biocompatibility and stability. Particle size decides the distribution of the NPs in the body. Larger NPs tend to be cleared faster in the blood, and more particles were distributed in the liver, lung and spleen. Smaller NPs (10–200 nm) with negative surface charge tend to accumulate in tumor and be cleared from the blood at a slower rate [37]. Drug loading capacity is directly correlated to the drug delivery efficiency. The stability of NPs is another important factor that affects NPs behavior in the blood stream [38]. Biodegradable and environment responsive NPs, whose degradation and subsequent drug release occur in pathological sites, become a subject of interest [39].

In our study, a pH sensitive DOX-hyd-PEG-FA NPs were successfully self-assembled into the core-shell structured micelles in aqueous solution, in which DOX molecules were packed in the core through intermolecular interaction between the anthracycline rings of DOX, while the folic acid ligands were exposed outside of the shell. The particle size and PDI value were stable in 5 days in PBS (pH = 7.4) at 37°C. The zeta potential of DOX-hyd-PEG-FA NPs and DOX-ami-PEG-FA NPs was −27.6 mV and −26.5 mV respectively. It was reported that negatively charged surface nanoparticles showed a reduced plasma protein adsorption and low rate of nonspecific cellular uptake [37], [40]. Meanwhile, the charged NPs can repel one another to overcome the natural tendency of aggregation of NPs [41]. Thus, DOX-hyd-PEG-FA NPs and DOX-ami-PEG-FA NPs had enough dispersion stability in the blood and were favorable for accumulation in the tumor tissue the by EPR effect.

It is very important to improve drug loading capacity and drug delivery efficiency for the active environment responsive NPs. The drug loading varied greatly in different drug delivery systems [42]. A tLyp-1-PEG-PLA nanoparticles loaded with paclitaxel was prepared through the emulsion/solvent evaporation technique, the drug loading was (1.43±0.10)%, and drug encapsulation efficiency was (47.5 ±2.4)% [43]. Guo X et al prepared a FA-PEG-PCL-hyd-DOX micelle. The DOX content in micelle was 4.57%. Moreover, it needs 8 steps reaction to synthesize FA-PEG-PCL-hyd-DOX [44]. In order to increase the drug delivery efficiency, DOX-hyd-PEG-FA NPs, DOX-hyd-PEG NPs, DOX-ami-PEG-FA NPs and DOX-ami-PEG NPs were prepared, and free DOX was encapsulated in NPs. There was not significant difference in the physical drug loading between different NPs. However, the total effective drug loading of DOX-hyd-PEG-FA NPs and DOX-hyd-PEG NPs were significant higher than that of DOX-ami-PEG-FA NPs and DOX-ami-PEG NPs. This was because large amount of DOX was released from DOX-hyd-PEG-FA and DOX-hyd-PEG polymer in acidic environment. Furthermore, there was not significant difference in zeta potential and particle size between DOX-hyd-PEG-FA NPs (−27.6±4.7 mV; 186±27 nm) and DOX-ami-PEG-FA NPs (−26.5±5.3 mV; 213±28 nm). Thus, the difference in anti-tumor efficacy between DOX-hyd-PEG-FA NPs and DOX-ami-PEG-FA NPs was resulted from the difference in the total effective drug loading. In a word, higher total effective drug loading was a very important advantage of DOX-hyd-PEG-FA NPs.

The faster release rate of DOX from the DOX-hyd-PEG-FA NPs in pH 5 medium (mimicking the endosomal/lysosomal environment of the tumor cells) was because of the acid-cleavable characteristics of the hydrazone linkage between the DOX and PEG, which resulted in the depolymerization of NPs in acidic medium. The slow DOX release rate of DOX-hyd-PEG-FA NPs observed at pH 7.4, mimicking the physiological conditions of the bloodstream, greatly reduced the chance of premature drug release in the blood circulation. The slow release rate of DOX from DOX-ami-PEG-FA NPs in acidic environment was because the amide bond between DOX and PEG was relative stable. Thus, DOX-ami-PEG-FA NPs were stable in acidic and neutral environment. It was relative difficulty to release DOX from DOX-ami-PEG-FA NPs in acidic and neutral environment.

After KB cells and A549 cells were incubated with free DOX for 4 h, the DOX mainly accumulated in the nucleus. Compared with the KB cells incubated with DOX-hyd-PEG NPs, the cells incubated with DOX-hyd-PEG-FA NPs showed more fluorescence in the nucleus and cytoplasm. Compared with KB cells, A549 cells incubated with DOX-hyd-PEG-FA NPs showed less fluorescence in the nucleus and cytoplasm. These results clearly indicated that the cellular uptake of DOX-hyd-PEG-FA NPs was facilitated by a folate receptor mediated endocytosis process. Furthermore, when DOX-hyd-PEG-FA NPs and DOX-ami-PEG-FA NPs were incubated with KB cells, they were localized in endolysosomes at 30 min. DOX-hyd-PEG-FA NPs showed a predominant nucleus distribution of DOX at 4 h. This was expected due to the dissociation of DOX-hyd-PEG-FA NPs in endolysosomes, subsequently faster release of DOX and led to a similar pattern of cellular distribution of free DOX by proton sponge effect [45]. However, DOX-ami-PEG-FA NPs were still found in endolysosomes after they were incubated with KB cells for 4 h. This was because DOX-ami-PEG-FA NPs were relative stable in acidic lysosomes.

The cytotoxicity of DOX-hyd-PEG-FA NPs and DOX-hyd-PEG NPs on A549 cells was much higher than that of DOX-ami-PEG-FA NPs and DOX-ami-PEG NPs respectively. This was due to the acidic sensitivity of DOX-hyd-PEG-FA NPs and DOX-hyd-PEG NPs, and more DOX was released in A549 cells. There was not significant difference in the viability when A549 cell was cultured with DOX-hyd-PEG-FA NPs and DOX-hyd-PEG NPs. This was because of the lack of FA receptors on A549 cells. For KB cells and HepG2 cells, the results showed that the cell viability in the presence of DOX-hyd-PEG-FA NPs was much lower than that of DOX-hyd-PEG NPs and free DOX, higher drug delivery efficiency of DOX-hyd-PEG-FA NPs was the reason for this observation. Meanwhile, the cytotoxicity of DOX-hyd-PEG-FA NPs against KB cells was inhibited by exogenous free FA. All the above results suggested that FA in DOX-hyd-PEG-FA NPs played an important role in enhancing the cytotoxic effect of DOX-hyd-PEG-FA NPs by binding itself with the overexpressed FA receptor, localized on the surface of the KB cell. Subsequently, it increased the intracellular uptake of DOX-hyd-PEG-FA NPs as a result of the receptor mediated endocytosis.

Compared with DOX, DOX-hyd-PEG NPs and DOX-ami-PEG-FA NPs, DOX-hyd-PEG-FA NPs significantly delayed the tumor growth. The enhanced *in vivo* antitumor effect of DOX-hyd-PEG-FA NPs was attributed to three factors: (1) passive targeting of DOX-hyd-PEG-FA NPs to tumor tissue; (2) active targeting of DOX-hyd-PEG-FA NPs to tumor cell overexpressing FA receptor; (3) high total effective drug loading and pH sensitive characteristics lead to the burst release of DOX from DOX-hyd-PEG-FA NPs in tumor cells. The passive targeting allowed DOX-hyd-PEG-FA NPs to accumulate in the tumor site, while the active targeting permitted it to be readily taken up by tumor cells at the tumor tissue.

The results of distribution experiment indicated that the level of DOX was above 15 µg/ g tissue in tumor tissue and was sustained for 24 h after DOX-hyd-PEG-FA NPs were administered. Longer exposure to DOX with the effective concentration leaded to a greater antitumor efficacy. Thus, the combined passive and active targeting effects, high total effective drug loading and pH sensitive were likely to act synergistically to increase the drug delivery efficiency of DOX-hyd-PEG-FA NPs, subsequently, delayed tumor growth. Finally, treatment with free DOX produced obvious side effects such as reduced activities and weight loss in animals. In contrast, we did not observe obvious side effects in mice treated with DOX-hyd-PEG-FA NPs, indicating that higher dose of DOX-hyd-PEG-FA NPs can be used to achieve a better therapeutic effect without introducing serious toxic effect.

In our lab, we previously synthesized FA-AMA-DOX and DOX-hyd-PEG conjugate to deliver DOX to tumor cell [18], [20]. The IC_50_ of FA-AMA-DOX and DOX-hyd-PEG conjugate on HepG2 cells was 5.0±0.8 mol/L and 7.6±0.9 µM respectively. The IC_50_ of DOX-hyd-PEG-FA NPs on HepG2 cells was 1.5±0.3 µmol/L. Moreover, compared with DOX-hyd-PEG conjugate, DOX-hyd-PEG-FA NPs delivered more DOX in tumor tissue, which resulted in a better antitumor activity in tumor bearing nude mice. The above results implied that DOX-hyd-PEG-FA NPs had more potential in targeted cancer therapy.
